# Psychische und neurokognitive Erkrankungen und Diabetes mellitus (Update 2023)

**DOI:** 10.1007/s00508-022-02117-9

**Published:** 2023-04-20

**Authors:** Heidemarie Abrahamian, Alexandra Kautzky-Willer, Angelika Rießland-Seifert, Diana Lebherz-Eichinger, Peter Fasching, Christoph Ebenbichler, Alexander Kautzky, Hermann Toplak

**Affiliations:** 1 Privates Institut für Medizin & NLP, Esteplatz 4/15, 1030 Wien, Österreich; 2 Gender Medicine Unit, Klinische Abteilung für Endokrinologie und Stoffwechsel, Universitätsklinik für Innere Medizin III, Medizinische Universität Wien, Wien, Österreich; 3 1. Psychiatrische Abteilung mit Zentrum für Psychotherapie und Psychosomatik, Klinik Penzing, Wien, Österreich; 4 Internistische Bettenstation mit Intermediate Care Unit, Klinik Penzing, Wien, Österreich; 5 Medizinische Abteilung für Endokrinologie, Rheumatologie und Akutgeriatrie, Klinik Ottakring, Wien, Österreich; 6 Universitätsklinik für Innere Medizin I, Medizinische Universität Innsbruck, Innsbruck, Österreich; 7 Klinische Abteilung für Sozialpsychiatrie, Medizinische Universitätsklinik für Psychiatrie und Psychotherapie, Wien, Österreich; 8 Klinische Abteilung für Endokrinologie und Diabetologie, Universitätsklinik für , Innere Medizin, Medizinische Universität Graz, Graz, Österreich

**Keywords:** Diabetes mellitus, Psychische Erkrankungen, Neurokognitive Erkrankungen, Depression, Angsstörungen, Schizophrenie, Diabetes mellitus, Mental disorders, Depression, Anxiety disorders, Schizophrenia

## Abstract

Diabetes mellitus ist häufig mit psychischen Erkrankungen assoziiert. Depressive Störungen kommen bei diabetischen Patient:innen doppelt so häufig vor wie in der nicht-diabetischen Population. Andere psychische Erkrankungen, die gehäuft mit Prädiabetes und Diabetes mellitus vorkommen, sind kognitive Dysfunktionen bis zur Demenz, auffälliges Essverhalten, Angststörungen, Schizophrenie, bipolare Störungen und Borderline-Persönlichkeitsstörungen. Die ungünstigen Auswirkungen dieser Koinzidenz auf den Stoffwechsel sind nachhaltig und manifestieren als schlechtere metabolische Kontrolle und vermehrte mikro- und makroangiopathische Komplikationen. Ziel dieses Positionspapieres ist die Sensibilisierung aller involvierten medizinischen Fachkolleg:innen sowie aller anderen mit dem Thema Diabetes befassten Berufsgruppen und Organisationen, um eine Intensivierung der komplexen therapeutischen Interventionen bei Patient:innen zu erreichen.

Positive Auswirkungen wären die Verringerung der Inzidenz von Diabetes mellitus bei Patient:innen mit psychischen Erkrankungen, sowie die Reduktion von Spätfolgen des Diabetes mellitus, insbesondere der kardiovaskulären Morbidität und Mortalität und eine verbesserte Lebensqualität der Betroffenen.

## Abstract (en)

Psychiatric disorders and psychological problems are common in patients with diabetes mellitus. There is a twofold increase in depression which is associated with suboptimal glycemic control and increased morbidity and mortality. Other psychiatric disorders with a higher incidence of diabetes are cognitive impairment, dementia, disturbed eating behavior, anxiety disorders, schizophrenia, bipolar disorders and borderline personality disorder. The coincidence of mental disorders and diabetes has unfavorable influences on metabolic control and micro- and macroangiopathic complications. Improvement of therapeutic outcome is a challenge in the modern health care system. The intentions behind this position paper are to rise awareness of this special set of problems, to intensify cooperation between involved health care providers and to reduce incidence of diabetes mellitus as well as morbidity and mortality from diabetes in this patient group.

## Einleitung

Zwischen Diabetes mellitus und bestimmten psychischen Erkrankungen besteht eine bidirektionale Beziehung. Depressionen, Angststörungen, Essstörungen und kognitive Defizite treten in der diabetischen Population gehäuft auf [1]. Die Zusammenhänge sind teilweise bekannt. Darüber hinaus können bestimmte psychische Erkrankungen wie Schizophrenie, schizotype und wahnhafte Störungen sowie bipolare Störungen zu einer erhöhten Inzidenz von Diabetes mellitus führen. Die Ursache dürfte in charakteristischen Besonderheiten der psychiatrischen Erkrankung und in potenziellen Nebenwirkungen von bestimmten Psychopharmaka begründet sein [1–3].

Menschen mit somatischer Morbidität in Kombination mit einer schweren psychischen Erkrankung weisen eine signifikant kürzere Lebenserwartung und eine zwei- bis dreifach erhöhte Mortalitätsrate im Vergleich zu psychisch gesunden, nur somatisch erkrankten Patient:innen auf [4]. Die Häufigkeit des Auftretens von psychischen Komorbiditäten ist bei Typ 1 Diabetes und Typ 2 Diabetes unterschiedlich und hängt unter anderem auch mit den jeweiligen pathophysiologischen und psycho-pathologischen Hintergründen zusammen [4].

In einer aktuellen groß angelegten Studie (Treatment Options for Type 2 Diabetes in Adolescents and Youth (TODAY2) study) wurde die Prävalenz von Depression, Essstörungen und verringerter gesundheitsbezogener Lebensqualität (Health related quality of life = HRQOL) bei jungen Erwachsenen mit Manifestation von Typ 2 Diabetes in der Jugend erhoben [5]. Des Weiteren wurde die longitudinale Assoziation der genannten psychischen Störungen mit Glykämie und Diabeteskomplikationen untersucht. Die Studienteilnehmer (*n* = 5514) wurden über einen definierten Zeitraum von 6 Jahren beobachtet. Das Lebensalter lag bei 21,7 ± 2,5 Jahren und die Diabetesdauer bei 8,6 ± 1,5 Jahren. Die Symptome von Depression und beeinträchtigter HRQOL waren initial häufig und nahmen über die Beobachtungsdauer signifikant zu (Depression von 14,0 % auf 19,2 %, *P* < 0,003; beeinträchtigte HRQOL von 13,1 % auf 16,7 %, *P* < 0,009). Depression und reduzierte HRQOL traten vermehrt bei Frauen und bei Patient:innen mit niedrigerem Familieneinkommen auf. Die Häufigkeit von Binge Eating Störungen blieb über den Beobachtungszeitraum konstant, Fälle von selbst berichteten Insulin Purging häuften sich. Symptome der Depression waren mit höheren HbA1c-Werten, mehr Hypertonie und ebenso mit einer Progression der Retinopathie assoziiert. Verringerte HRQOL wies eine Assoziation mit höherem BMI, höherem systolischen Blutdruckwert und ebenso mit Progression der Retinopathie auf. Die TODAY2 Studie zeigt eindrucksvoll die Häufigkeit von psychischen Beeinträchtigungen bereits bei jungen Patient:innen mit in der Jugend manifestiertem Typ 2 Diabetes, sowie die ungünstigen Auswirkungen auf den Verlauf des Diabetes mellitus [5].

Der Einfluss psychischer Erkrankungen auf die Qualität der Stoffwechselkontrolle bzw. auf kardiovaskuläre Risikofaktoren bei Diabetes mellitus ist in der Regel ungünstig und signifikant und beeinflusst die Entwicklung von mikro- und makrovaskulären Spätschäden [1, 4].

Ziel dieses Positionspapieres ist sowohl die Sensibilisierung aller involvierten medizinischen Fachkolleg:innen sowie sonstiger mit diesem Thema befasster Berufsgruppen und Organisationen als auch die Intensivierung der komplexen therapeutischen Interventionen bei Patient:innen mit der Koinzidenz Diabetes mellitus und psychische Erkrankung. Analog den Anforderungen des publizierten Position Statements der Amerikanischen Diabetes Gesellschaft sollte auch in Österreich eine umfassende psychosoziale Betreuung in die Diabetesbehandlung integriert werden und für alle Menschen mit Diabetes mellitus zugänglich gemacht werden [1].

Positive Auswirkungen der Umsetzung dieses Positionspapieres wären die Verringerung der Inzidenz von diabetischen Neuerkrankungen bei psychisch kranken Menschen, die konsekutive Reduktion von Spätfolgen bei an Diabetes erkrankten Menschen, insbesondere der kardiovaskulären Morbidität und Mortalität, sowie eine verbesserte Lebensqualität der Betroffenen.

## „Diabetes Distress“

Die Anforderungen und Belastungen, die die Erkrankung Diabetes mellitus durch das erforderliche Selbstmanagement mit sich bringt, werden individuell unterschiedlich erlebt und bewertet und können zu einer erheblichen kognitiven und emotionalen Stressbelastung mit psychischer Beeinträchtigung führen. Das Auftreten von „diabetes distress“ („Diabetes spezifischer Stress“) wird bei Typ 1 und Typ 2 Diabetes beschrieben. Psychische Symptome entwickeln vor allem Patient:innen, die durch das Selbstmanagement des Diabetes mellitus kognitiv und emotional überfordert sind. Häufig besteht ein Zusammenhang mit ungünstigen Lebensumständen [1]. Die diagnostischen Kriterien einer Depression werden dabei nicht erfüllt, obwohl teilweise überlappende Symptome bestehen. Da sich Diagnostik, Verlauf und Therapie wesentlich voneinander unterscheiden, ist eine klare Differenzierung der Begriffe „Depression“ und „Diabetes spezifischer Stress“ bzw. der klinischen Bilder erforderlich [6].

Die Prävalenz von Diabetes spezifischem Stress liegt mit 18–45 % aller an Typ 1 oder Typ 2 Diabetes mellitus erkrankten Personen relativ hoch [1]. Da negative Auswirkungen auf HbA1c, Selbsteffektivität, Lebensqualität und Therapieadhärenz beschrieben werden, ist einerseits die Diagnostik wichtig, andererseits zeitnah zur Diagnosestellung die Teilnahme an einer Diabetesschulung erforderlich [1, 5, 7]. In dieser Schulung soll der Umgang mit körperlichen, psychischen und sozialen Anforderungen, die mit der Erkrankung einhergehen, erlernt werden. Darüber hinaus ist zu prüfen, ob der Patient/die Patientin über ausreichende personelle, soziale und ökonomische Ressourcen verfügt und diese auch nutzen kann. Ein bestimmtes Ausmaß an individueller Stressresilienz ist erforderlich, um den eigenverantwortlichen Umgang mit der Erkrankung praktizieren zu können. Für den Behandler stellt sich das Bild von hohem diabetesbezogenem, nicht bewältigbarem Stress häufig auch als mangelnde Therapieadhärenz dar, wobei Überlastung und Überforderung des Patienten/der Patientin dabei im Vordergrund stehen. Ist die Einschätzung bezüglich aktivierbarer Ressourcen und Stressresilienz negativ, sollte eine psychotherapeutische Intervention als begleitende Maßnahme zur Seite gestellt werden.

Für die Diagnostik sind folgende Instrumente gut validiert und zu empfehlen: Problem Areas In Diabetes (PAID) umfasst 20 Fragen, die sowohl emotionalen Stress als auch spezifische Belastungen des Diabetes erfassen [1, 8]. Eine deutschsprachige Version steht zur Verfügung. Die Diabetes Distress Scale (DDS) umfasst 17 Fragen und erlaubt durch die Einbeziehung von 4 Bereichen wie emotionale Belastung, interpersonelle Belastung, Arzt-bezogener Stress und Therapie-bezogener Stress eine umfassende Beurteilung mit einer soliden Bewertungsmöglichkeit. Eine validierte Übersetzung in die deutsche Sprache liegt aktuell nicht vor [1, 9].

## Depression

Die Beziehung zwischen Depression und Diabetes mellitus ist bidirektional und bildet damit eine Nahtstelle zwischen den medizinischen Fachdisziplinen [1, 10–14].

Nach der ICD-10-Klassifikation unterscheidet man bei Patient:innen mit depressiven Symptomen leichte, mittelgradige und schwere depressive Episoden, weiters rezidivierende depressive Störungen, anhaltende affektive Störungen und andere seltenere affektive Störungen.

Das Risiko für das Erkranken an einer Depression ist für Patient:innen mit Diabetes mellitus etwa doppelt so hoch im Vergleich zu einer nicht-diabetischen Kontrollgruppe [15]. In einer Metaanalyse wird die Häufigkeit der Depression bei Patient:innen mit Diabetes mellitus Typ 2 mit 17,6 % angegeben. Das entspricht einer deutlich höheren Prävalenzrate als bei Patient:innen ohne Diabetes mellitus (9,8 %) [15]. Wie auch in der nicht-diabetischen Population sind Frauen häufiger betroffen als Männer (23,8 % versus 12,8 %).

Als ursächlich werden psychische Belastungen durch die Herausforderungen einer chronischen Erkrankung sowie gemeinsame pathophysiologische Mechanismen diskutiert. Die publizierten Daten der Diabetes Attitudes Wishes and Needs Study 2 (DAWN2) bestätigen bei Patient:innen mit Diabetes mellitus individuell empfundene Belastungen bezogen auf Lebensqualität, Selbstmanagement, Einstellungen zur Krankheit sowie Defizite durch mangelhafte soziale Unterstützung über mehrere Länder hinaus [16].

Subklinische Inflammation und Dysfunktion der Hypothalamus-Hypophysen-Nebennieren (HHN)-Achse bzw. Aktivierung des sympathischen Nervensystems werden im Rahmen der Depressionsentstehung bei Diabetes mellitus und bei anderen chronischen somatischen Erkrankungen als kausal diskutiert [12, 15].

Die Interferenz der Depression mit der Qualität der Stoffwechseleinstellung dürfte von der Anzahl und Schwere der psychischen Symptome linear abhängig sein [15, 17]. Daten der ACCORD (Action to Control Cardiovascular Risk in Diabetes)-Studie zeigen, dass bei diabetischen Patient:innen mit Depression die Mortalität in Abhängigkeit vom Schweregrad der Depression signifikant um das 1,8- bis 2,2-Fache erhöht ist [11].

Ein Screening auf das Vorliegen einer Depression ist insbesondere bei Patient:innen mit problematischer Diabeteseinstellung sinnvoll und zielführend. Hierfür hat sich der Zwei-Fragen-Test bewährt [18]:

1. Gab es in den letzten 4 Wochen eine Zeitspanne, während der Sie sich nahezu jeden Tag niedergeschlagen, traurig und hoffnungslos fühlten?
2. Gab es in den letzten 4 Wochen eine Zeitspanne, während der Sie das Interesse an Tätigkeiten verloren haben, die Ihnen sonst Freude machten?

Werden beide Fragen bejaht und wird ein durchgehender Zeitraum von mindestens zwei Wochen angegeben, sollte weiter untersucht werden, ob eine behandlungsbedürftige Depression vorliegt. Allerdings ist zu beachten, dass zwar die Sensitivität dieses Screening-Tests hoch, jedoch die Spezifität bei positivem Ergebnis niedrig ist, sodass neben einer ausführlichen Anamnese eine weiterführende Diagnostik, z. B. mittels Beck Depression Inventory, Hospital Anxiety and Depression Scale (HADS-D) und WHO-Five-Well-Being Index (WHO‑5, http://who-5.org/) erforderlich ist [19, 20].

Therapeutisch sollten Maßnahmen ergriffen werden, die sowohl die depressiven Symptome als auch die metabolische Situation auf Grund des Diabetes mellitus günstig beeinflussen. Dabei können unterschieden werden: 1. psychologische Interventionen (z. B. Psychoedukation – siehe auch Kapitel Schulung innerhalb dieses Positionspapieres, Kognitiv-Behaviorale Therapie), 2. (psycho)pharmakologische Behandlung sowie 3. Methoden zur Modifikation des Lebensstils.

### 1. Psychologische Interventionen

Hierzu ist ein systematisches Review über 13 klinische Studien zur Behandlung von komorbid vorliegender Depression und Diabetes zu nennen [21, 22]. Die angewendeten Interventionen waren höchst unterschiedlich in Bezug auf Zeitausmaß, Art der Anwendung (z. B. persönlich, telefonisch, internetbasiert), Inhalt (z. B. Kognitiv-behaviorale Therapie, Psychoedukation), intervenierende Berufsgruppe (z. B. Ärzt:innen, Pflegekraft, Psychotherapeut:innen). Kognitiv behaviorale Therapie (CBT) zeigte die höchste Evidenz, in mehreren Studien konnten moderate bis große antidepressive Effekte nachgewiesen werden. Diese Effektivität lässt sich noch steigern, wenn CBT mit weiteren psychologischen Techniken kombiniert wird, wie z. B. Stress-Management-Strategien, Coping-Skills-Training, Case-Management u. a. Eine solche Vorgangsweise ist in Bezug auf die positive Beeinflussung von depressiven Symptomen der alleinigen Verordnung von Antidepressiva überlegen [21].

Die Studienergebnisse bezüglich positiver Effekte von psychologischen Interventionen auf die glykämische Kontrolle sind sehr heterogen, es gibt keine klare Evidenz. In der Mehrzahl der Untersuchungen ließen sich jedoch positive Effekte nachweisen [22].

### 2. (Psycho)pharmakologische Behandlung

Bei mittelgradigen und schweren depressiven Episoden wird einerseits die Beiziehung von Fachärzt:innen für Psychiatrie sowie die Verordnung von antidepressiver Medikation empfohlen. Es ist zu beachten, dass das Nebenwirkungsprofil von antidepressiven Medikamenten je nach Substanzklasse sowie auch innerhalb derselben verschieden ist und eine an das Patient:innenprofil angepasste Auswahl des Therapeutikums erfordert [23, 24]. Häufige, für Patient:innen mit einer chronischen Stoffwechselerkrankung besonders relevante Nebenwirkungen umfassen gastrointestinale Symptome wie Übelkeit, Erbrechen und Diarrhoe, Sedierung, Agitation, Schlafstörung, Gewichtszunahme, Erhöhungen der Blutzuckerwerte, Verlängerung der QT-Zeit (Citalopram und Escitalopram), Hyperprolaktinämie, Sexualfunktionsstörungen, Thrombozyten-aggregationshemmung und andere [24, 25].

In rezenten systematischen Reviews und Metaanalysen lässt sich, wie für die allgemeine Bevölkerung, die Effektivität aller häufig verordneten Antidepressiva belegen [23]. Für selektive Serotonin Reuptake-Inhibitoren (SSRI) ergibt sich eine moderate Effektivität im Vergleich zu Placebo sowie eine günstige Beeinflussung des HbA1C, wobei letztere nicht generalisierbar erscheint [21, 22].

Antidepressiva, welche eine signifikante Gewichtszunahme induzieren (z. B. Paroxetin, Mirtazapin, Trizyklika), könnten die glykämische Kontrolle durch Steigerung der Insulinresistenz verschlechtern. Serotonin Noradrenalin Reuptake-Inhibitoren (SNRI) sind gegenüber SSRI wegen des geringeren Risikos der Nebenwirkungen sexuelle Dysfunktion und Gewichtszunahme möglicherweise die zu bevorzugenden Substanzen. Eine Studie mit dem selektiven SNRI Milnacipran zeigte gute Effekte sowohl auf die depressive Symptomatik als auch sekundär auf Stoffwechselparameter ohne Auswirkungen auf die Sexualfunktion [13]. Agomelatin weist möglicherweise ein besonders günstiges Nebenwirkungsprofil mit Gewichtsneutralität auf [26]. Bupropion als Dopamin- und Noradrenalin-Wiederaufnahmehemmer ist mit Gewichtsabnahme assoziiert und hat keine Nebenwirkungen auf die Sexualfunktion, jedoch ein gering erhöhtes, dosisabhängiges Risiko für zerebrale Krampfanfälle [21, 22, 25].

Der Einsatz antidiabetischer Medikation gegen depressive Symptome bei insulinresistenten Patient:innen fußt auf dem theoretischen Wissen, dass kognitive und affektive Störungen durch zentrale Insulinresistenz gefördert werden können [21, 27]. Die Substanz Pioglitazon, die Insulinresistenz reduziert, hat in zwei randomisierten kontrollierten Studien antidepressive Effekte gezeigt. Zusätzlich werden intranasales Insulin und Liraglutid in Bezug auf mögliche antidepressive Effekte untersucht [21, 28].

### 3. Lebensstilmodifikation

Der Versuch einer positiven Beeinflussung des Lebensstils der Betroffenen ist entscheidend sowohl für die Besserung einer Depression als auch des Diabetes mellitus. Körperliche Aktivität, gesunde Ernährung, Schlafhygiene und Tabakabstinenz verbessern das psychische und das medizinische Outcome.

Als Ausdruck der bidirektionalen Beziehung zwischen Diabetes mellitus und Depression zeigte sich für depressive Patient:innen das Risiko an Diabetes mellitus Typ 2 zu erkranken in etwa verdoppelt [14, 29, 30]. Als Ursache dafür werden ebenso inflammatorische Phänomene und eine Dysfunktion der HHN-Achse als biologische Verbindungen zwischen Depression und Diabetes diskutiert. Die Therapie gestaltet sich wie oben ausgeführt.

Auswirkungen von Antidepressiva auf den Stoffwechsel sind in Tab. 1 dargestellt.

**Table 1:** 

	Substanz	Gewicht	Plasmaglukose
Trizyklische Antidepressiva	Amitriptylin, Nortriptylin	±	±
MAO-Inhibitoren	Phenelzin, Tranylcypromin	+ + +	−
	Moclobemid	0/−	NA
SSRI	Citalopram, Fluoxetin, Paroxetin, Sertralin, u. a.	±	0/−
SNRI	Duloxetin, Venlafaxine, Milnacipran	0/−	0
Andere	Bupropion	0/−	0
	Mirtazapin	+ +	0/+

Gewicht: + + + deutliche, + + moderate, 0/+ minimale bis keine Gewichtszunahme. 0/− minimale bis keine Gewichtsabnahme. ± uneinheitliche Angaben

Plasmaglukose: 0/+ nicht eindeutig nachgewiesener Anstieg, 0/− nicht eindeutig nachgewiesene Reduktion, − Reduktion, ± unterschiedliche Angaben zu Auswirkungen auf die Plasmaglukose. 0 keine Auswirkungen

*NA* no data available

## Angststörungen

Angststörungen werden nach ICD-10-Klassifikation in phobische Störungen (Agoraphobie mit und ohne Panikstörung, soziale Phobie, spezifische (isolierte) Phobien), Panikstörung, generalisierte Angststörung, Angst und depressive Stimmung gemischt, Zwangsstörung sowie Reaktionen auf schwere Belastungen (akute Belastungsreaktion, posttraumatische Belastungsstörung) und Anpassungsstörungen eingeteilt. Angststörungen treten bei Patient:innen mit Diabetes häufiger auf als in der nicht-diabetischen Population, wobei lediglich für generalisierte Angststörungen und für Panikstörungen ausreichende Daten vorliegen [32, 33]. Speziell diabetesbezogene Ängste, wie verstärkte und übermäßige Angst vor Hypoglykämien und vor Spätschäden, müssen in der Anamnese berücksichtigt werden, da sie zu ungünstigen Auswirkungen auf die metabolische Kontrolle führen können.

Die Diagnostik der Angststörungen ist komplex und erfordert die Anwendung strukturierter klinischer Interviews und psychometrischer Fragebögen. Für die Therapie ist ein Gesamtbehandlungsplan zu entwerfen, der in der Regel Psychotherapie und medikamentöse Ansätze umfasst. Als Medikamente kommen je nach Erkrankung und eventueller Komorbidität in erster Linie Antidepressiva, aber auch Pregabalin und adjuvant Benzodiazepine zum Einsatz. Bei letzteren ist die Gefahr der Abhängigkeitsentwicklung zu beachten, eine Dauerverordnung ist nicht indiziert.

## Essstörungen

Die Klassifikation von Essstörungen erfolgt nach dem ICD-10-Code in Anorexia nervosa, Bulimia nervosa, Essattacken bei anderen psychischen Störungen, nicht näher bezeichnete Essstörungen und andere. Die häufig diskutierte Annahme der erhöhten Prävalenz von Anorexie und Bulimie konnte bei Diabetes mellitus Typ 1 bis dato in Studien nicht bestätigt werden. Allerdings kommt gestörtes Essverhalten, ohne die Diagnosekriterien für eine Essstörung zu erfüllen, in Kombination mit Diabetes mellitus Typ 1 häufiger vor als in der nicht-diabetischen Population [34, 35].

Für Patient:innen mit Diabetes mellitus Typ 2 liegen in der Literatur Hinweise vor, die auf ein vermehrtes Auftreten von Bulimie, Binge Eating Disorder und andere Essstörungen wie Night Eating Disorder schließen lassen. Allerdings ist die Datenlage inkonsistent [34, 35].

Zu beachten ist in jedem Fall, dass die Komorbidität Essstörung zu einer Verschlechterung der Stoffwechselsituation, zu einem vermehrten Auftreten von Retinopathie und Neuropathie und bei Menschen mit Diabetes mellitus Typ 1 zu einer erhöhten Frequenz von Krankenhausaufenthalten wegen diabetischer Ketoazidose führt [35].

Insbesondere bei jüngeren Patient:innen mit instabiler Metabolik und signifikanten Gewichtsschwankungen sollte an Bulimie, Binge-Eating-Disorder oder Insulin-Purging, das als Mittel zur Gewichtsreduktion eingesetzt wird, gedacht werden.

Die Diagnostik erfolgt mittels Anamnese sowie ergänzend durch strukturierte klinische Interviews und/oder Fragebögen bzw. gibt es für Patient:innen mit Diabetes mellitus adaptierte diagnostische Instrumente [35].

Die Therapie der Wahl bei Essstörungen besteht in psychotherapeutischen Interventionen wie z. B. systemische Familientherapie, Verhaltenstherapie, Gesprächstherapie oder psychodynamische Therapie, eingebettet in einen Gesamtbehandlungsplan.

## Borderline-Persönlichkeitsstörungen

Der Verlauf einer kombinierten Erkrankung von Diabetes mellitus und Borderline-Persönlichkeitsstörung wird durch die z. T. erhebliche Beeinträchtigung der Impulskontrolle und die Schwierigkeiten bei der Beziehungsgestaltung geprägt, was sich auch auf die medizinische Betreuung auswirkt. Die Patient:innen fallen z. B. durch besonders schlechte Therapieadhärenz, selbstschädigendes Verhalten oder Substanzmissbrauch auf. In einer aktuellen Studie lag die altersstandardisierte Prävalenz von metabolischem Syndrom bei Borderline-Patient:innen 2‑fach höher als in der psychisch gesunden Kontrollgruppe (23,3 % vs. 10,6 %) [36]. Die Prävalenz von Diabetes mellitus wird mit 9 % angegeben [37]. Die Ursachen dafür dürften multifaktoriell sein, wobei auch teilweise die verordneten Psychopharmaka zur Diabetesmanifestation beitragen können.

Die wichtigste Basis für das weitere ärztliche Management besteht in der Herstellung einer tragfähigen Arzt-Patienten-Beziehung.

## Schizophrene und bipolare Erkrankungen

Die Prävalenz von Typ 2 Diabetes ist bei Patient:innen mit schizophrenen, schizoaffektiven und bipolaren Erkrankungen 2‑ bis 3‑fach höher als in der Normalbevölkerung. Patient:innen mit einer schweren psychischen Störung erkranken an Diabetes mellitus im Durchschnitt 10–20 Jahre früher als die psychisch gesunde Bevölkerung. Die Ursachen dafür dürften nach aktuellem Wissensstand multifaktoriell sein, wobei folgende zu nennen sind: krankheitsspezifische Faktoren, niedriger sozioökonomischer Status, in dessen Folge ungesunder Lebensstil (Rauchen, ungesunde Ernährung, Bewegungsmangel) [4, 38, 39], ungenügende medizinische Versorgung und nicht zuletzt ungünstige Nebeneffekte bestimmter Psychopharmaka [4, 25]. Die meisten Studien zeigen, dass metabolische Auffälligkeiten bald nach Behandlungsbeginn mit Neuroleptika auftreten, die bei ca. 12–13 % dieser Patient:innen zu einem manifesten Diabetes mellitus führen [36]. Ebenso kommt es zu einer Kumulation weiterer kardiovaskulärer Risikofaktoren wie Adipositas, Dyslipidämie und metabolischem Syndrom. In den Clinical Antipsychotic Trials of Intervention Effectiveness (CATIE) wurde gezeigt, dass ein Drittel der Patient:innen mit Schizophrenie signifikante unbehandelte metabolische und kardiovaskuläre Risikofaktoren aufwies [38].

Neuere wissenschaftliche Daten weisen auf einen gemeinsamen Vulnerabilitätsfaktor für Diabetes mellitus, metabolisches Syndrom und Schizophrenie hin. Insbesondere wird eine aberrante Aktivierung des Monozyten/Makrophagen-Systems mit abnormer Bildung von Zytokinen und Adipokinen diskutiert [40].

In einer schwedischen Register-Studie wurde bei 1.736.281 Teilnehmern untersucht, wie viele von ihnen im Untersuchungszeitraum von max. 19 Jahren (median 10,6 Jahre), eine Schizophrenie, eine schizoaffektive Psychose und Typ 1 oder Typ 2 Diabetes manifestierten [41]. Dabei zeigte sich, dass Individuen mit Schizophrenie ein deutlich höheres Risiko für Typ 1 Diabetes aufwiesen als Individuen ohne Schizophrenie (HR 2,84). Für schizoaffektive Psychosen konnte diese Koinzidenz nicht bestätigt werden. Das Risiko, Typ 2 Diabetes zu entwickeln war für beide genannten psychiatrischen Krankheitsbilder signifikant erhöht, für Menschen mit Schizophrenie HR (95 % CI): 13,98 (8,70–22,46), *p* < 0,0001 und für Menschen mit schizoaffektiver Psychose HR (95 % CI): 14,27 (7,36–27,70), *p* < 0,0001. Bei den vielfältigen pathophysiologischen Mechanismen, die für die häufige Koinzidenz von Diabetes mellitus und Schizophrenie sowie schizoaffektive Psychose verantwortlich sind, spielen sowohl genetische Faktoren als auch Umweltfaktoren eine Rolle. Es ist anzunehmen, dass ausgehend von gemeinsamen GEN-Funktionen im Gehirn oder Pankreas, bestimmte Kaskaden und Mechanismen aktiviert werden, die für die Manifestation der jeweiligen Komorbidität verantwortlich sind [41].

Die größte Herausforderung bei schwerverlaufenden Erkrankungen und in akuten Krisen ist die mangelnde bis fehlende Krankheits- und Behandlungseinsicht der Betroffenen. Ziel ist immer die Herstellung einer tragfähigen Arzt-Patienten-Beziehung, auf deren Basis alle weiteren diagnostischen und therapeutischen Maßnahmen aufgebaut und koordiniert werden können. Dazu gehören im Sinne eines Gesamtbehandlungsplans eine möglichst individuell abgestimmte psychopharmakologische Behandlung, eine somatisch-medizinische Diagnostik und Betreuung sowie verschiedene unterstützende sozio- und psychotherapeutische Maßnahmen.

## Kognitive Störungen

Die Klassifikation der Demenz erfolgt nach dem ICD-10-Code in Demenz bei Alzheimer Krankheit, vaskuläre Demenz, Demenz bei andernorts klassifizierten Erkrankungen, nicht näher bezeichnete Demenz und andere Demenzformen. Sowohl mit Typ 1 Diabetes als auch mit Typ 2 Diabetes werden strukturelle zerebrale Veränderungen mit klinischen Manifestationen, wie kognitive Defizite und Demenz, assoziiert. In großen, epidemiologischen Studien wurde gezeigt, dass vaskuläre und Alzheimer Demenz bei Patient:innen mit Diabetes gehäuft auftreten. Bei Typ 2 Diabetes ist das Risiko für eine vaskuläre Demenz 2‑ bis 4‑fach erhöht und das Risiko an einer Alzheimer Demenz zu erkranken 1,5- bis 2‑fach erhöht [42]. Der kausale Zusammenhang mit Diabetes mellitus ist wahrscheinlich durch das Auftreten von multiplen Risikofaktoren wie Glykämie, Hypertonie, Dyslipidämie, kardiale Erkrankungen u. a. zu erklären. Persistierende Hyperglykämie scheint eine wichtige Rolle bei der Entwicklung einer zerebralen Dysfunktion zu spielen [43, 44]. Passagere kognitive Einschränkungen durch akute Blutzuckerveränderungen, wie Hypo- und Hyperglykämie, sind bekannt und werden in diesem Kapitel nicht näher bearbeitet.

Die Heterogenität sowohl der diagnostischen Methoden als auch der untersuchten Populationen erschwert konklusive Aussagen über die Assoziation zwischen Typ 1 Diabetes und der Entwicklung von kognitiven Störungen. In der prospektiven Längsschnittstudie Epidemiology of Diabetes Interventions and Complications (EDIC-Nachfolgestudie des DCCT) über einen Zeitraum von 18 Jahren zeigten sich keine substanziellen kognitiven Defizite [44]. In einer Studie zu morphologischen Veränderungen des Gehirns wurden 95 jugendliche Patient:innen mit Typ 1 Diabetes und 49 Geschwister als Kontrollpersonen, im Alter zwischen 7–17 Jahren untersucht. Die Frequenz von schweren Hypoglykämien und HbA1c-Werte als Ausdruck der Glykämie-Exposition wurden dokumentiert und eine entsprechende Stratifizierung vorgenommen. Patient:innen mit höherer Frequenz von schweren Hypoglykämien wiesen ein größeres Hippocampus-Volumen auf als Patient:innen mit einer niedrigeren Frequenz von schweren Hypoglykämien [45].

In einer Substudie der ACCORD-Studie, der ACCORD Memory in Diabetes (MIND)-Study, wurde der Zusammenhang zwischen Glykämie und kognitiver Funktion an einer großen Population mit Typ 2 Diabetes untersucht [46]. Der Glykämie-Status wurde mittels Nüchternblutzucker und HbA1c ermittelt. Für die Beurteilung der kognitiven Funktion wurde der DSST-Score (Digit Symbol Substitution-Test) und für die Evaluierung der Hirnstruktur wurde eine Magnetresonanztomographie durchgeführt. Obwohl das totale Hirnvolumen in der Gruppe mit intensivierter Diabetestherapie signifikant höher war, zeigte sich kein Unterschied im DSST-Score zwischen intensivierter und Standardtherapie.

Für die Praxis relevant ist die ungünstige Auswirkung der kognitiven Defizite auf das erforderliche Selbstmanagement bei Diabeteserkrankungen. Für die Diagnostik eignen sich unter anderem folgende etablierte Tests: Demenz-Detektions-Test (DemTect), Mini-Mental-Status-Test (MMST) und Uhrentest.

## Psychopharmaka und Stoffwechsel

Für die erhöhte Morbidität und Mortalität von psychisch kranken Patient:innen werden auch die teils stark ausgeprägten metabolischen Nebenwirkungen zahlreicher Psychopharmaka (viele Antipsychotika, manche Antidepressiva, Phasenprophylaktika) verantwortlich gemacht ([4, 25, 26]; Tab. 2). Zu den wichtigsten metabolischen Nebenwirkungen zählen Gewichtszunahme, eine Erhöhung des Risikos an Diabetes zu erkranken und die Entwicklung eines atherogenen Lipidprofils.

**Table 2:** 

Substanz	Gewichtszunahme	DM-Risiko	Dyslipidämie Risiko
Clozapine	+ + +	+	+
Olanzapin	+ + + (5,0 kg)	+	+
Risperidon	+ + (2,0 kg)	DI	DI
Quetiapine	+ +	DI	DI
Aripiprazol	0/+	−	−
Ziprasidon	0/+ (0,6 kg)	−	−

+ minimal, + + moderat, + + + stark steigernder Effekt. 0/+ möglicher gering steigernder Effekt. − kein Effekt

Zahlen in Klammern: Gewichtszunahme in Kilogramm

*DI* Datenlage inkonklusiv

Generell liegt ausreichende Evidenz für den Zusammenhang zwischen antipsychotischen Medikamenten und erhöhtem Risiko für Diabetes mellitus vor, allerdings ist dieses Risiko im Vergleich zu den traditionellen Diabetes-Risikofaktoren klein. In einer Metaanalyse wurde das Diabetesrisiko durch die atypischen im Vergleich zu den konventionellen Antipsychotika mit 1,32 (95 % CI 1,15–15,1) angegeben [3, 4]. Die Evidenz für unterschiedliche Effekte der verschiedenen atypischen Antipsychotika ist weniger konklusiv, wobei das Ausmaß der Gewichtszunahme eine Rolle spielen dürfte. In der aktuellen Literatur finden sich nur wenige prospektive Studien zu diesem Thema. In einem systematischen Review der vorhandenen prospektiven randomisierten Studien zu der Frage: „Blutzucker und Schizophrenie“, finden sich keine konsistenten signifikanten Unterschiede [47]. Die kritische Frage: „Welcher Anteil des Risikos für die Manifestation metabolischer Störungen ist den Medikamenten zuzuordnen?“ kann bis dato nicht zufriedenstellend beantwortet werden. Die einseitige Assoziation zwischen kurzfristiger Gewichtszunahme unter Therapie mit atypischen Antipsychotika und dem Diabetesrisiko ignoriert alle anderen Ursachen von Gewichtszunahme bei Patient:innen mit schizophrenen Erkrankungen, wie Umgebungsfaktoren oder spezielle Verhaltensmuster und Genetik (s. oben). In den meisten Studien sind häufige Komorbiditäten von schizophrenen Störungen wie Alkohol- oder Drogenmissbrauch bzw. -abhängigkeit und Depression nicht in die Überlegungen einbezogen [48].

Die Mechanismen, durch welche Antipsychotika Gewichtszunahme induzieren, sind tatsächlich weitgehend ungeklärt. Zahlreiche Hypothesen beschäftigen sich mit Effekten auf den Hypothalamus, einem Antihistamineffekt, einem sedierenden Effekt der Psychopharmaka mit Tagesmüdigkeit und daraus resultierender verminderter körperlicher Bewegung, einem Effekt auf die Leptinkonzentration und andere [4, 25, 49].

Auch manche sogenannte Phasen-Prophylaktika wie z. B. Lithiumsalze, Carbamazepin und Valproinsäure können Auswirkungen auf den Stoffwechsel in Form von Gewichtszunahme und Hyperglykämie haben. Sie werden einerseits zur Akutbehandlung und Rückfallprophylaxe bei bestimmten affektiven Erkrankungen (bipolare Störung, unipolare Depression) oder schizoaffektiven Erkrankungen eingesetzt, andererseits werden manche von ihnen auch bei anderen Indikationen erfolgreich verwendet (z. B. Epilepsie).

Eine medikamentös bedingte ausgeprägte Gewichtszunahme kann den Therapieerfolg entscheidend beeinflussen. Auf sozialer Ebene kann es durch die ästhetische Beeinträchtigung zu einer Stigmatisierung der Patient:innen kommen, die eine weitere Belastung darstellt. Daneben erfordern viele metabolische Nebenwirkungen zusätzliche therapeutische Maßnahmen, was in Summe zu Frustration, schlechter Therapieadhärenz oder Therapieabbruch führen kann. Abschließend ist noch zu erwähnen, dass sowohl typische als auch atypische Antipsychotika eine hohe Potenz für die Entwicklung einer Hyperprolaktinämie haben [24, 25].

## Compliance – Adhärenz – Konkordanz

Bei der Behandlung von Patient:innen mit Diabetes mellitus wurde bereits vor geraumer Zeit festgestellt, dass das Konzept der „Compliance“ (Befolgung, Fügsamkeit) nicht geeignet ist bzw. nicht funktioniert [50]. Fünfundneunzig Prozent der Zeit ihres Lebens behandeln diese Patient:innen ihre Krankheit selbst und entwickeln daher ihre ganz persönlichen Strategien des Umgangs. Besser geeignet ist daher das Prinzip der „Adhärenz“. Patient:innen sollen für ein sinnvolles Vorgehen und Verhalten gewonnen werden [51]. Das in der Sozialpsychiatrie bewährte Modell des Empowerments ist auch in der Behandlung von diabetischen Patient:innen sehr geeignet [52]. Ziel ist es, die Patient:innen in die Lage zu versetzen bzw. dabei zu unterstützen, dass sie selbst ihr Leben und auch ihre gesundheitlichen Probleme meistern können. Nur wenn es gelingt, eine „gemeinsame Landkarte“ zu entwerfen, wenn die Ziele von Arzt und Patient:innen übereinstimmen, wird die Zusammenarbeit gut funktionieren und in der Folge die Diabeteseinstellung zufriedenstellend sein. In diesem Sinne wird in der Literatur auch der Begriff „Concordance“ verwendet [53].

## Diabetes und Selbstgefährdung

Generell ist bei allen Patient:innen mit Diabetes mellitus und psychischer Komorbidität mit einer potenziellen chronischen Selbstgefährdung zu rechnen, v. a. durch mangelnde Therapieadhärenz. Ebenso ist eine erhöhte Suizidgefährdung anzunehmen, es gibt jedoch keinen Konsens bezüglich der Höhe des Risikos, z. B. stellte eine britische Studie fest, dass Patient:innen mit Typ 1 Diabetes eine 11-fach erhöhte Rate von Suizidversuchen mit Insulin aufweisen [54]. Eine Untersuchung von Anfragen an eine Vergiftungszentrale ergab, dass ca. 0,16 % aller Anfragen Überdosierungen mit Insulin betrafen, davon 90 % in suizidaler oder parasuizidaler Absicht [55]. Immerhin beträgt die Letalität der Verabreichung von sehr hohen Insulindosen in suizidaler Absicht 3–10 %, als Dauerschaden kann es durch schwere Unterzuckerung zu einer Enzephalopathie kommen. Depressive Patient:innen mit Diabetes mellitus und Insulintherapie müssen daher ärztlich besonders sorgfältig geführt werden. Dazu gehören nicht nur eine genaue Anamnese mit Erfassung von Risikofaktoren, sondern auch eine psychiatrische Mitbehandlung und Psychotherapie sowie bei Bedarf Unterstützung durch andere Personen, z. B. zur Insulinverabreichung.

## Diabetesschulungen und psychotherapeutische Begleitung

Sorgfältiges Selbstmanagement ist bei Patient:innen mit Diabetes mellitus besonders wichtig, um Akutkomplikationen, wie Hyper- und Hypoglykämie und Langzeitschäden, wie Mikro- und Makroangiopathie zu vermeiden. Der Erwerb von Kenntnissen über Ursachen, Verlauf und Therapie der Krankheit und von bestimmten Fertigkeiten ist Voraussetzung für die effektive und dauerhafte Umsetzung von therapeutischen Maßnahmen im persönlichen Alltag. Eine Diabetesschulung, die die besonderen Gegebenheiten bei psychisch kranken Patient:innen berücksichtigt, ist daher ein wesentlicher Grundstein für die Behandlung. Zu den besonderen Problemen bei dieser Patientengruppe zählen Gewichtszunahme durch bestimmte Psychopharmaka, hohe Tagesmüdigkeit, unstrukturierter Tagesablauf, wenig Krankheitseinsicht und andere, die alle die Umsetzung gesundheitsfördernder Maßnahmen behindern können.

In der rezenten Literatur finden sich wenige Daten zum Thema Diabetesschulung bei Patient:innen mit psychischer Komorbidität. Eine ältere Untersuchung zeigt, dass die Kombination aus Diabetesschulung und kognitiver Verhaltenstherapie bei Patient:innen mit Typ 2 Diabetes und Depression signifikant bessere Auswirkungen auf den HbA1c-Wert zeigte als Diabetesschulung allein [56]. In einer rezenten Studie konnte bei diabetischen Patient:innen mit Depression ein ähnlicher integrativer Ansatz bestätigt werden [57].

Obwohl die positiven Auswirkungen von kognitiver Verhaltenstherapie und anderen Psychotherapieformen auf depressive Symptome außer Frage stehen, sind Auswirkungen von psychotherapeutischer Begleitung allein (ohne strukturierte Diabetesschulung) auf Stoffwechselparameter weniger gut belegt. Studien zu dieser Fragestellung weisen in der Regel kleine Fallzahlen und kurze Dauer auf [58–60]. In einem systematischen Review bestätigte sich, dass nicht pharmakologische Therapien, wie psychologische Behandlung und Psychotherapie, zwar die depressiven Symptome reduzierten, jedoch häufig nicht zu einer Verbesserung der Metabolik führten [61].

Ein ideales Modell für psychisch kranke Patient:innen würde aus einer Kombination von strukturierter Diabetesschulung (mit Adhärenztraining) und Psychotherapie bestehen, wobei durch diesen integrativen Ansatz sowohl eine Besserung der Depressionssymptome als auch ein besseres Verständnis von Diabetes mellitus mit Steigerung der Therapieadhärenz erreicht werden könnten.

## Empfehlungen

Um bei Patient:innen mit der Koinzidenz somatische und psychische Morbidität das metabolische und kardiovaskuläre Risiko zu reduzieren, sollten definierte Screening- und Monitormaßnahmen wahrgenommen werden [1, 4, 7, 25, 62, 63]:

1. Die Erhebung des psychosozialen Status sollte als essenzieller Bestandteil in die Betreuung diabetischer Patient:innen eingeführt werden. Dieser umfasst Lebenssituation und Lebensqualität, Stimmung, Einstellung zur Erkrankung, Erwartungen in Hinblick auf Krankheitsverlauf und Therapie, Verfügbarkeit von Ressourcen und andere.
2. Darüber hinaus sollten alle Patient:innen mit Diabetes mellitus einmal jährlich auf das Vorliegen folgender psychischer Erkrankungen gescreent werden: Diabetes-spezifischer Stress („diabetes distress“), Depression, Angststörung und Essstörung. Ein jährliches Screening auf kognitive Dysfunktion und Demenz empfiehlt sich bei Patient:innen über 65 Jahre.
3. Bei psychisch kranken Patient:innen mit antipsychotischer oder antidepressiver Therapie ist die Erhebung kardiovaskulärer und metabolischer Risikofaktoren zu Beginn der Therapie und im Verlauf sinnvoll [24, 25]. Folgende Screening- und Monitoring-Empfehlung hat sich in der Praxis bewährt: zumindest einmal jährliches Screening in Hinblick auf die Risikofaktoren Diabetes mellitus, Dyslipidämie, viszerale Adipositas und Hypertonie. Messungen von Blutdruck, Bauchumfang und Gewicht sollten einen Monat nach Beginn der Therapie mit Psychopharmaka und anschließend in Abständen von 3 bis 6 Monaten durchgeführt werden. Die Messung des HbA1c ist in 3‑monatigen Abständen sinnvoll. Bestimmungen von Prolaktin im Serum sind bei für Hyperprolaktinämie prädisponierender Psychopharmaka-Therapie ein bis zweimal jährlich empfehlenswert ([25]; Tab. 3). Schulungen für diese spezielle Patientengruppe sollten angeboten werden.

**Table 3:** 

	Therapiebeginn	Woche 4	Woche 12–26	Jährlich
Vorerkrankungen	X	–	–	–
Familienanamnese	X	–	–	–
Bewegung	X	–	–	X
Ernährung	X	–	–	X
Nikotin	X	–	–	X
Beratung RF	X	X	X	X
Gewicht	X	X	X	X
BU	X	X	X	X
Blutdruck	X	X	X	X
BZ nüchtern	X	X	X	X
HbA1c^a^	X	–	X^a^	X^a^
Lipide	X	X	X	X
Prolaktin	X	–	–	X

^a^In 3‑monatigen Abständen

Die prophylaktische Etablierung einer niedrig dosierten Metformin-Therapie ist bei ungünstiger Entwicklung in Richtung metabolisches Syndrom unter Psychopharmaka-Therapie in Erwägung zu ziehen, bedeutet jedoch einen „Off-Label“-Einsatz.

4. Diese Screening- und Monitorleitlinie sowie therapeutische Interventionen wie Umstellung der Ernährung, regelmäßige körperliche Bewegung, Motivation zu Tabak- und Alkohol-Karenz sollen in interdisziplinärer Kooperation zwischen Allgemeinmedizinern, Internisten-Endokrinologen und Psychiatern umgesetzt werden.
5. Bei Menschen mit der Koinzidenz von Typ 2 Diabetes und psychischer bzw neurokognitiver Erkrankung ist die antihyper-glykämische Therapie so weit wie möglich entsprechend den Leitlinien der ÖDG umzusetzen. [s. Kapitel Antihyperglykämische Therapie bei Diabetes mellitus Typ 2] Insgesamt ist die Studienlage zu antihyperglykämischer Therapie in dieser Patientengruppe spärlich. Wie bereits oben beschrieben, kann bei viszeraler Adipositas und anderen Parametern des metabolischen Syndroms unter antipsychotischer Therapie der frühe Einsatz von Metformin in Erwägung gezogen werden. Die gute Wirkung von GLP1-Analoga auf Blutzucker und Gewicht bei Menschen mit Typ 2 Diabetes und psychischer Komorbidität wird in mehreren Studien bestätigt [64, 65]. Für SGLT2-Hemmer liegen für die Anwendung bei psychisch kranken Menschen keine Daten vor. Die Frage nach einer möglicherweise kumulativen Inzidenz von Ketoazidosen durch die Kombination von SGLT2-Hemmern und Antipsychotika (beide Pharmaka weisen eine erhöhte Inzidenz für Ketoazidosen auf) kann derzeit nicht beantwortet werden [66, 67]. DPP4-Hemmer und Glitazone können entsprechend den Leitlinien der ÖDG eingesetzt werden. Bei der Komorbidität Typ 1 Diabetes und psychische Erkrankung ist zu bedenken, dass komplexe Insulintherapien zu Überforderung und hyperglykämischen Entgleisungen führen können.

## Interessenkonflikt

H. Abrahamian hat von folgenden Unternehmen, die auch fördernde Mitglieder der ÖDG sind, Forschungsunterstützungen und/oder Honorare erhalten: Amgen, Boehringer Ingelheim, Novo Nordisk, sanofi-aventis, Servier. Zusätzlich gibt H. Abrahamian an, dass für sie persönlich kein Interessenkonflikt vorliegt. A. Kautzky-Willer hat von folgenden Unternehmen, die auch fördernde Mitglieder der ÖDG sind, Forschungsunterstützungen und/oder Honorare erhalten: AstraZeneca, Bayer Health Care, Böehringer-Ingelheim, Bristol-Myers Squibb, GlaxoSmithKline Pharma, Eli Lilly, Merck Sharp & Dohme, Novartis, Novo Nordisk, Roche, sanofi-aventis. A. Rießland-Seifert hat von folgenden Unternehmen, die auch fördernde Mitglieder der ÖDG sind, Forschungsunterstützungen und/oder Honorare erhalten: AstraZeneca, Bristol-Myers Squibb, Eli Lilly, Lundbeck. P. Fasching hat von folgenden Unternehmen Forschungsunterstützungen und/oder Honorare erhalten: Abbott, AstraZeneca, Bayer Health Care, Boehringer Ingelheim, Bristol-Myers Squibb, Daiichi-Sankyo, Germania Pharmazeutika, GlaxoSmithKline Pharma, Eli Lilly, Merck Serono, Merck Sharp & Dohme, Novartis, Novo Nordisk, Pfizer, Roche, sanofi-aventis, Servier, Takeda. Zusätzlich gibt P. Fasching an, dass für ihn persönlich kein Interessenkonflikt vorliegt. Er legt offen, dass er in diesem Zeitraum von den genannten Firmen Honorare für Vorträge und Consulting erhalten hat bzw. Fortbildungsunterstützung im Rahmen der (dienst)rechtlichen Rahmenbedingungen bezogen hat (Einladung zu Kongressen) bzw. Klinische Studien als PI mit einzelnen Firmen durchgeführt hat bzw. durchführt. C. Ebenbichler hat von folgenden Unternehmen, die auch fördernde Mitglieder der ÖDG sind, Forschungsunterstützungen und/oder Honorare erhalten: Amgen, AstraZeneca, Boehringer Ingelheim, Novartis, Novo Nordisk, Sanofi-aventis. H. Toplak hat von folgenden Unternehmen, die teilweise fördernde Mitglieder der ÖDG sind, Forschungsunterstützungen und/oder Honorare erhalten: Amgen, Boehringer Ingelheim, Bristol-Myers Squibb, Eli Lillly, Merck Sharp & Dohme, Novartis, Novo Nordisk, Pfizer, Sanofi-aventis. A. Kautzky und D. Lebherz-Eichinger geben an, dass kein Interessenkonflikt besteht.
